# Smooth ZnO:Al-AgNWs Composite Electrode for Flexible Organic Light-Emitting Device

**DOI:** 10.1186/s11671-017-1841-2

**Published:** 2017-01-25

**Authors:** Hu Wang, Kun Li, Ye Tao, Jun Li, Ye Li, Lan-Lan Gao, Guang-Yong Jin, Yu Duan

**Affiliations:** 1 College of Science, Changchun University of Science and Technology, Changchun, 130012 China; 2 State Key Laboratory on Integrated Optoelectronics, College of Electronic Science and Engineering, Jilin University, Jilin, 130012 China

**Keywords:** Silver nanowires, Al-doped ZnO, Atomic layer deposition, Transparent conductive electrode, Organic light-emitting device

## Abstract

The high interest in organic light-emitting device (OLED) technology is largely due to their flexibility. Up to now, indium tin oxide (ITO) films have been widely used as transparent conductive electrodes (TCE) in organic opto-electronic devices. However, ITO films, typically deposited on glass are brittle and they make it difficult to produce flexible devices, restricting their use for flexible devices. In this study, we report on a nano-composite TCE, which is made of a silver nanowire (AgNW) network, combined with aluminum-doped zinc oxide (ZnO:Al, AZO) by atomic layer deposition. The AgNWs/AZO composite electrode on photopolymer substrate shows a low sheet resistance of only 8.6 Ω/sq and a high optical transmittance of about 83% at 550 nm. These values are even comparable to conventional ITO on glass. In addition, the electrodes also have a very smooth surface (0.31 nm root-mean-square roughness), which is flat enough to contact the OLED stack. Flexible OLED were built with AgNWs/AZO electrodes, which suggests that this approach can replace conventional ITO TCEs in organic electronic devices in the future.

## Background

Transparent conducting electrodes (TCEs) have been widely used in photo-electronic devices such as thin film transistors, thin film solar cells, and organic light-emitting devices (OLEDs) [1–3], due to their excellent optical and electrical properties, in particular, indium tin oxide (ITO) [4]. However, ITO has important disadvantages. It is increasingly expensive and hard to produce in a sustainable manner because of a limited supply of indium. In addition, its fragility is a mismatch with the demands by flexible electronics, the next generation light source, and display technology [5–7]. Recently, a variety of materials have been investigated as substitutes for ITO, including conjugated polymers [8], carbon nanotubes (CNTs) [9], graphene [10], silver nanowires (AgNWs) [11, 12], and metal oxides such as zinc oxide (ZnO) [13, 14]. Among these alternatives, AgNWs, which uses one-dimensional nano-sized silver as the primary material, are most promising because of their outstanding electrical, optical and mechanical properties, Furthermore, AgNWs films can be deposited using a low-cost solution process, Dong-Seok Leem et al. [15] fabricated P3HT:PCBM OSCs using AgNWs as the lower electrode, leading to high PCEs of up to 3.5%. Nonetheless, pure AgNWs films have several drawbacks, such as a high surface roughness, a large junction resistance, and a small contact area [16, 17]. The buried-layer structure, which comprises a AgNWs film and a metal − oxide layer, has been suggested as an alternative to overcome these issues [18–20]. Sanggil Nam et al.[21] had reported that AgNWs electrode embedded in NOA63 has an ultra-smooth surface with a root-mean-square (RMS) of 0.4 nm and Rpv of 4.557 nm.

In our previous work, we have prepared AgNWs/ZnO electrode in which ZnO as a conductive-bridging for AgNWs was deposited by low temperature (60 °C) ALD [22], but the sheet resistance was higher than AgNWs. In this work, we reported an AgNWs/AZO hybrid film on a photopolymer NOA63 substrate, where AZO was deposited 6 nm by ALD. AZO reduced the junction resistance immensely by strengthening the junction connection of the AgNWs. It also increased the contact area by filling open spaces between the AgNWs. Macroscopically, AZO reduced the sheet resistance of the AgNWs film. It cut down the height difference between the AgNWs and the substrate, and smoothed out the surface roughness. A composited TCE with a low sheet resistance of 8.6 Ω/sq, a transmittance of 83% at 550 nm, and less than 1 nm surface roughness was obtained.

## Methods

**Fig. 1:**
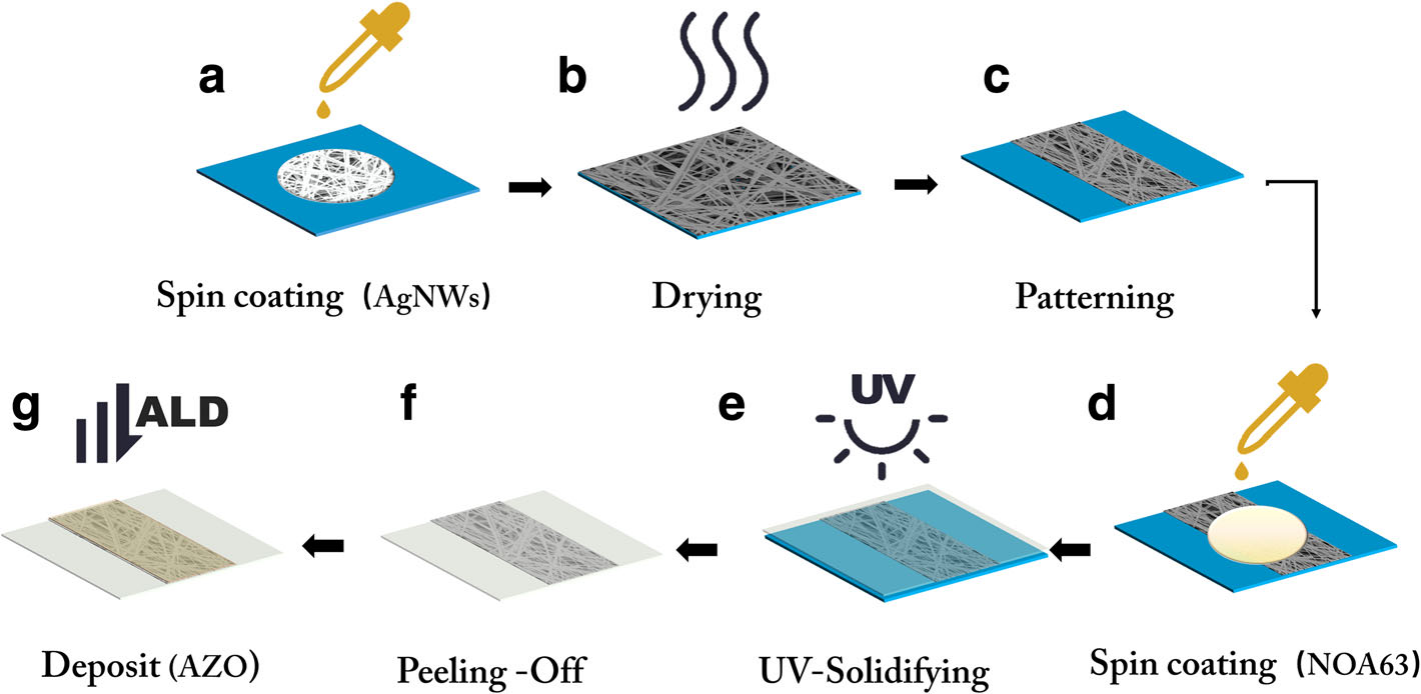
The flow-process of fabricating flexible electrode. **a** Dropping AgNWs solution and spin coating. **b** Drying in the oven at 150 °C. **c** Patterning electrode. **d** Dropping NOA63 and spin coating. **e** Exposing under ultraviolet light with 300–370 nm wavelength for 4 min. **f** Peeling the NOA63 off. **g** Depositing AZO by ALD

The procedures to fabricate the electrodes are shown in Fig. 1. Isopropanol-based solutions of AgNWs (XFNano Company, NanJing, China) with a ratio of length to diameter (70 nm × 100 μm) were shaken in an ultrasonic machine for 5 min and deposited on a clean silicon substrate via spin coating at 6000 rpm for 30 s. We chose the large length diameter ratio of silver nanowires. This was because at the same concentration the longer nanowires form films with better conductivity and a better surface topography. Then, the AgNWs film was dried in an oven at 150 °C for 10 min in order to remove solvent and to soften the AgNWs, followed by the “Patterning”, which means wiping off the AgNWs on both sides of silicon. An alcohol cotton ball was used to wipe off AgNWs and only leave the middle part (1 cm wide). Because the anode and cathode of our organic light-emitting device are crossed, they will be connected directly if we did not wipe off AgNWs on both sides. After patterning, NOA63 (Norland Optics, Norland Products, Cranbury, NJ, USA), a type of photoresist, was deposited on the AgNWs film by spin coating with 300 rpm for 15 s first, and then 600 rpm for 15 s. The samples were exposed to ultraviolet light at a 300–370-nm wavelength for 4 min for solidifying, and a flexible AgNWs-only electrode was obtained.

**Fig. 2:**
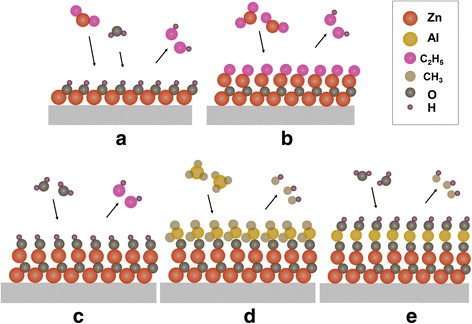
The schematics of the reaction process in ALD. **a** Reaction DEZ and H_2_O reacting. **b** Accessing DEZ, reacting and discharging C_2_H_6_. **c** Accessing H_2_O, reacting and discharging C_2_H_6_. **d** Accessing TMA, reacting and discharging CH_4_. **e** Accessing H_2_O, reacting and discharging CH_4_

The AZO was grown with a Lab Nano 9100 ALD system (Ensure Nanotech Inc. Beijing, China) at 150 °C which could soften the AgNWs and made them interconnect better, and this temperature has little effect on the NOA63 and the electrode. The equation shown in Fig. 2 indicates that diethyl zinc ((C_2_H_5_)_2_Zn, DEZ) and tri-methyl aluminum (Al(CH_3_)_3_, TMA), respectively, react with H_2_O in the reaction chamber with a constant 20 sccm flow of high purity N_2_, which carries and purges the residual precursor vapors at 0.1 Torr pressure. High purity nitrogen was used as the purging gas; the purging time was long enough to exhaust gaseous residual products, including gaseous precursors without reactions and gaseous reaction products. So, there are almost no residual products in ALD process. As shown in Fig. 2a, DEZ and H_2_O react on the substrate surface, and –C_2_H_5_ react with H_2_O and generate one layer of –OH functional groups on the top of the sedimentary layer. Then, to access the DEZ, as shown in Fig. 2b, one of the –C_2_H_5_ on DEZ react with –OH and generate C_2_H_6_; this process is performed according to Eq. (1). For the next reaction, shown in Fig. 2c considering to Eq. (2), to access H_2_O, the –C_2_H_5_ react with H_2_O and generate –OH functional groups on the top of the sedimentary layer. This is the same for the process in Fig. 2a. Equations (1) and (2) represent a reaction cycle, and then ZnO is grown layer by layer. The principle of growing Al_2_O_3_ is similar to ZnO, to access TMA, −CH_3_ on TMA react with –OH and generate CH_4_, then to access H_2_O, react with –CH_3_, and completes doping of Al—see Fig. 2d, (e) and Eqs. (3) and (4) [23].

1 $$ \mathrm{Surf}-\mathrm{Z}\mathrm{n}(OH)+\mathrm{Z}\mathrm{n}{\left( C{H}_2 C{H}_3\right)}_2\to \mathrm{Surf}-\mathrm{Zn}\mathrm{OZn}\left( C{H}_2 C{H}_3\right)+{\mathrm{C}}_2{H}_6 $$

2 $$ \mathrm{S}\mathrm{u}\mathrm{r}{\mathrm{f}}^{\prime }-\mathrm{Z}\mathrm{n}\left( C{H}_2 C{H}_3\right)+{H}_2\mathrm{O}\to \mathrm{S}\mathrm{u}\mathrm{r}{\mathrm{f}}^{\prime }-\mathrm{Z}\mathrm{n}(OH)+{\mathrm{C}}_2{H}_6 $$

3 $$ \mathrm{S}\mathrm{u}\mathrm{r}{\mathrm{f}}^{\prime }-\mathrm{Z}\mathrm{n}(OH)+\mathrm{A}\mathrm{l}{\left( C{H}_3\right)}_3\to \mathrm{S}\mathrm{u}\mathrm{r}{\mathrm{f}}^{\prime }-\mathrm{Zn}\mathrm{OAl}{\left( C{H}_3\right)}_2+ C{H}_4 $$

4 $$ \mathrm{S}\mathrm{u}\mathrm{r}{\mathrm{f}}^{{\prime\prime} }-\mathrm{A}\mathrm{l}\left( C{H}_3\right) + {H}_2\mathrm{O}\to \mathrm{S}\mathrm{u}\mathrm{r}{\mathrm{f}}^{{\prime\prime} }-\mathrm{A}\mathrm{l}(OH)+\mathrm{C}{H}_4 $$

ZnO is accessed with the 0.03 s pulses, and after waiting 30 s, H_2_O is accessed to oxidize DEZ. Then it takes 100 s to purge completely both the C_2_H_6_ and the residual gas. The growing of Al_2_O_3_ follows a cycle sequence: 0.02 s TMA dose, 30 s waiting, 0.02 s H_2_O dose, 100 s purge. According to the parameters above, we deposited 3 nm AZO as follows: 15 cycles ZnO/1 cycle Al_2_O_3_/15 cycles ZnO, and 6 nm of 20 cycles ZnO/1 cycle Al_2_O_3_/20 cycles ZnO/1 cycle Al_2_O_3_/20 cycles ZnO, and 9 nm of 22 cycles ZnO/1 cycle Al_2_O_3_/22 cycles ZnO/1 cycle Al_2_O_3_/22 cycles ZnO/1 cycle Al_2_O_3_/22 cycles ZnO. The doping ratio of Al was optimized at 3 at.% for all depositions.

Finally, we fabricated green light OLEDs with the AgNWs/AZO composite anode. The structure of the OLED was 2 nm MoO_3_ grown on anode as a hole-buffer layer; 30 nm thick 4,4′,4″-tris (N-3-methylphenyl-N-phenylamino)-triphenylamine (m-MTDATA) as hole-injection layer; 20 nm thick N,N’-biphenyl N,N’-bis (1-naphenyl)-[1,1′-biphenyl]-4,4′-diamine (NPB) as a hole transport layer; 30 nm thick tris-(8-hydroxyquinoline) aluminum (Alq_3_) doped with 1% 10-(2-benzothiazolyl)-2,3,6,7-tetrahydro-1,1,7,7-tetramethyl-1H,5H,11H-(1)-benzopyropyrano(6,7-8-I,j) quinolizin-11-one (C545T) as the main light-emitting layer; 20 nm of Alq_3_ as the electron transport layer; 0.5 nm of Liq and 100 nm Al as the cathode. The active area is 0.1 cm^2^. An Agilent B2902A source meter and a Minolta luminance meter LS-110 were used to measure the current density–voltage–luminance (I–V–L) characteristics of the flexible devices.

## Results and Discussions

**Fig. 3:**
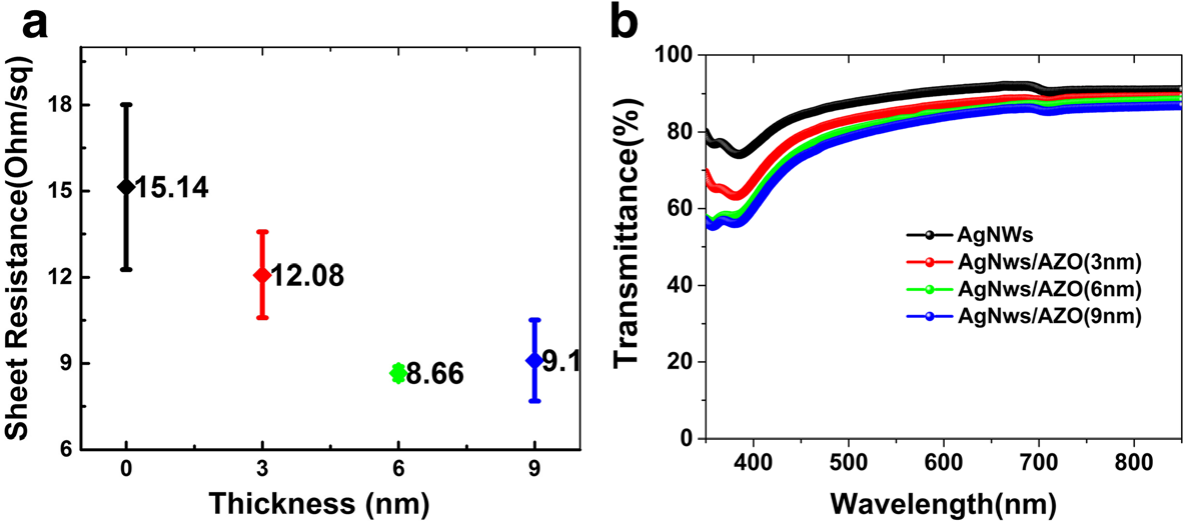
**a** The averages and error values of sheet resistance of AgNWs-only and AgNWs with 3, 6, and 9 nm AZO electrodes. **b** The transmittance of AgNWs-only and AgNWs with 3, 6, and 9 nm AZO electrodes in visible light range

In order to compare the optical and electrical properties, we fabricated samples of AgNWs electrodes with different thicknesses of AZO, and we prepared an AgNWs-only sample for reference. Figure 3a shows the effect for composite electrodes with different thicknesses of AZO layers on the sheet resistance. The AgNWs-only film had floating values on for Rs from 12.7 to 18.0 Ω/sq because the rough surface of the electrode produces random connection with the four-point probe. The sample with 3, 6, and 9 nm AZO had 12.0 ± 1.5, 8.66 ± 0.4, and 9.1 ± 1.4 Ω/sq, respectively [24]. For organic device electrodes, the optical transmittance is an important parameter, because it directly affects the light output coupling. Figure 3b shows transmittances of different electrode grown with 0, 3, 6, 9 nm thickness AZO in the range of visible light wavelength. At 550 nm, the transmittance of the AgNWs-only electrode was 89%. In this work, the transmittance depended on both the concentration of AgNWs solution and the spin-coating speed. Here, we selected a 5 mg/ml concentration and 6000 rmp speed considering to our previous research [22]. The transmittance of the electrode for 3 nm AZO was 85% at 550 nm wavelength, for 6 nm AZO it was 83%, and for 9 nm AZO it was 81%. With the increase of the thickness of AZO, the transmittance decreased. For the electrode, both the excellent conductivity and optical transmittance are necessary; however, the two properties usually mutually restrict; a thicker film tends to have a better conductivity but lower transmittance. In order to optimize the trade-off between transmittance and sheet resistance, the following figure of merit (Φ_FM_) [25], as defined by Haacke, was introduced into our work:

$$ {\varPhi}_{\mathrm{FM}}=\frac{T^{10}}{R_S}, $$

where *T* is the transmittance at a wavelength of 550 nm and *Rs* is the sheet resistance (Ω/sq). The Φ_FM_ values calculated by this formula are expected to be better the bigger they are. Here, we chose the four-test point method for each sample and calculate Φ_FM_. In Table 1, the Φ_FM_ of the three different samples was summarized. Φ_FM_ of AgNWs electrode with 3 nm AZO was 16.406, with 6 nm it was 17.917 which is the highest value and with 9 nm it was 13.360. Although 3 nm AZO affected on transmittance less than thicker AZO, it had poor connectivity for AgNWs on substrate surface. This was not as good as the 6 and the 9 nm AZO layer. The reasons are that 3 nm was too thin to form a continuous AZO layer, so the reduction of Rs was limited. For the 9 nm AZO modified layer, we obtained a relatively low Rs of 9.1 Ω/sq; however, the AZO film has a low transmittance compared to the AgNWs-only electrode. If AZO is grown thicker, the Rs reduction of the composite electrode will slow down because AZO as a semiconductor material itself is lacking carriers. The Rs was higher than for the AgNWs film according to the report by Manuela Göbelt, where the pure AZO film has an 18 times higher Rs (368 Ω/sq) than the heated AgNWs network [26], while its transmittance will decrease. Therefore, we optimized the thickness of the modified AZO layer of 6 nm. It was not only perfectly connected AgNWs on the surface of substrate and decreased sheet resistance of AgNWs film, but also had up to 83% light transmittance.

**Table 1:** Characteristic values of for all samples with different thicknesses for the AZO modified layer

Structure	Thickness of AZO (nm)	Average of Rs (Ω/sq)	Transmittance at 550 nm	Figure of merit (Φ_FM_ × 10^−3^)
AgNWs/AZO	3	12.0	85%	16.406
AgNWs/AZO	6	8.6	83%	17.917
AgNWs/AZO	9	9.1	81%	13.360

**Fig. 4:**
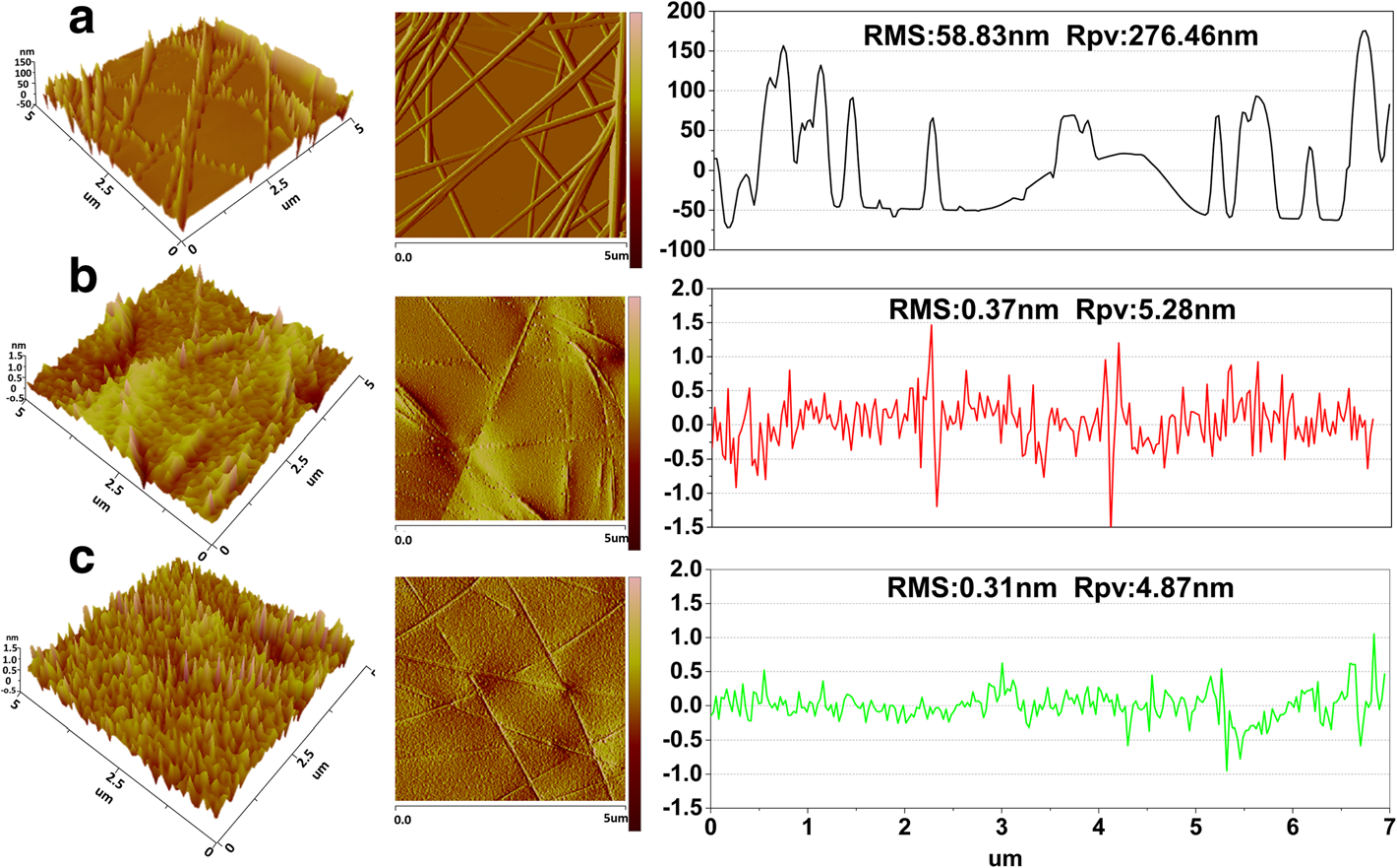
Three-dimensional AFM images in tapping mode, planar diagram and height line profiles of three samples **a**. The AgNWs spin-coated on a silicon chip. **b** AgNWs embedded in NOA63. **c** AgNWs/AZO (6 nm) embedded NOA63 (5 × 5 um)

Furthermore, the AZO modified layer also contributed to the improved surface smoothness. Figure 4 shows the image taken with atomic force microscope (AFM) in tapping mode, including a three-dimensional diagram, planar diagram, and the height line profiles of the AgNWs on a silicon chip, as well as AgNWs embedded in NOA63, and AgNWs embedded NOA63 covered with AZO. The roughness of the directly prepared AgNWs on the silicon substrate is very high, and the maximum can reach several hundred nanometers. A RMS of 58.83 ± 0.05 nm and a maximum peak-to-valley (Rpv) of 276.46 ± 0.05 nm were measured. It was easy to cause short circuits in the devices because the total thickness of the organic layers of the devices is below 100 nm. The AgNWs embedded in the NOA63 film showed a surface with RMS of 0.37 ± 0.02 nm and Rpv of 5.28 ± 0.03 nm. This shows clearly that the NOA63 not only fills the open spaces, but also covers the protruding thorns and crossing knots of AgNWs. The use of NOA63 is a common method, but after peeling NOA63 off, the surface of electrode is not smooth enough. After the fabrication of the device, the first layer of MoO_3_ was only 2 nm thick, so the roughness of surface of electrode must decrease. In this work, we introduced an AZO layer and measured the surface roughness of the AgNWs embedded NOA63 with AZO. The obtained RMS is 0.31 ± 0.01 nm, while the Rpv is 4.87 ± 0.02 nm. Both values are lower than the values for AgNWs embedded NOA63. This indicates the modified AZO layer also contributes significantly to the improved surface smoothness [18, 27]. It also suggests the surface of the AgNWs electrode was not smooth enough alter peel off; the reasons may be attributed to the viscosity of NOA63. When NOA63 flows into the vacancy of the AgNWs, the high viscosity prevented NOA63 from permeating through and bonding with the AgNWs. Therefore, gaps between AgNWs and NOA63 substrate are present on nanoscale, see Fig. 4b. The AZO film improved the surface roughness further by filling these gaps. It thus created a smoother surface for the organic layer [28]. As we can see in Fig. 4c, a homogeneous surface was obtained by depositing the AZO film on AgNWs embedded on the NOA63 substrate.

**Fig. 5:**
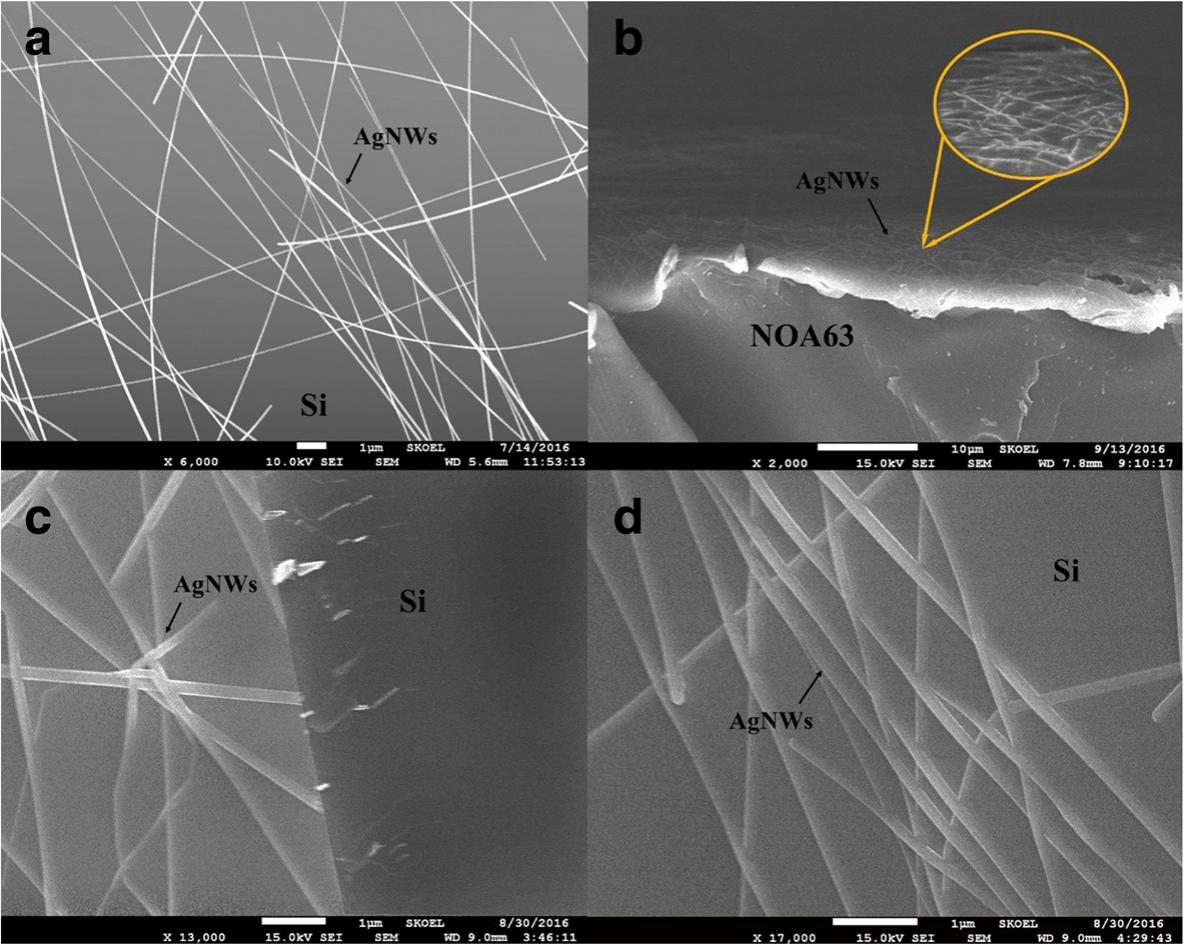
SEM micrographs of (**a**) AgNWs with large length to diameter ratio. **b** AgNWs embedded in NOA63. **c,d** AgNWs covered by AZO on silicon chip

Figure 5a shows the SEM image of AgNWs on a silicon substrate. As we know, the photoelectric properties of AgNWs-based transparent electrodes depend on the density of meshwork. With increasing density, the conductivity increases due to the better connection between AgNWs, while transmittance decreases because of the reflection of AgNWs mesh. AgNWs with a large length to diameter ratio can effectively ensure high electrical conductivity, but it also reduces the reflection to balance its light transmittance. The plane graph of AgNWs embedded in NOA63 is shown in Fig. 5b. The AgNWs becomes blurred in enlarged view, which suggests that AgNWs was embedded in NOA63. This was also in accordance with the AFM result shown in Fig. 4b. Figure 5c and d presents a profile and plane photograph of the AgNWs/AZO composite electrode on a silicon substrate, the shadows on the AgNWs reveal an AZO film with a thickness of 6 nm blanketed AgNWs that form a core-shell structure and enhanced between the AgNWs [26].

**Fig. 6:**
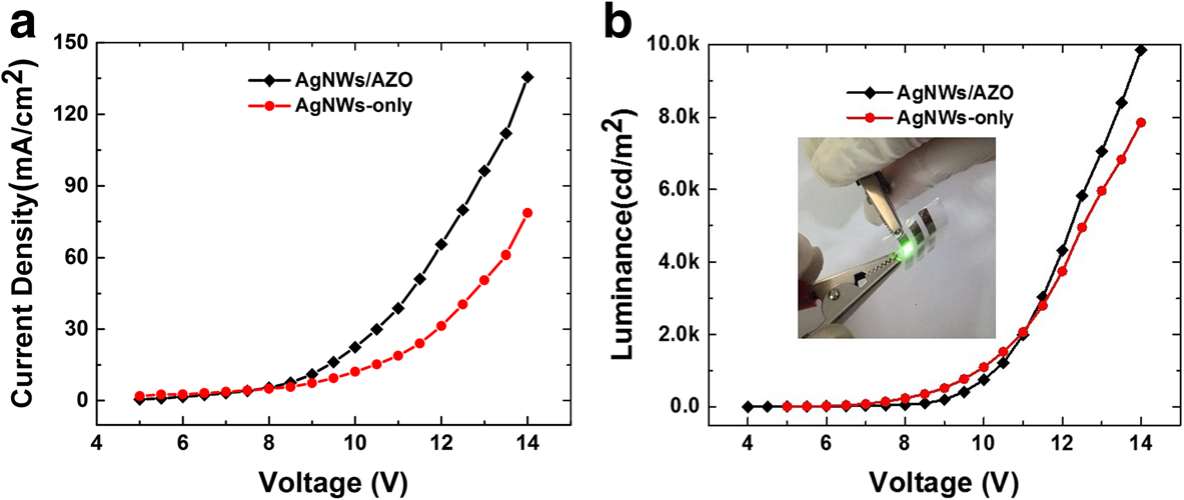
I–V–L characteristic of the green light-emitting device. **a** Current density–voltage characteristics of the AgNWs/AZO (6 nm) composite electrode and the referential AgNWs-only electrode. **b** Luminance–voltage characteristics of the AgNWs/AZO(6 nm) composite electrode and the AgNWs-only reference electrode

Figure 6 shows I–V–L characteristic of the green organic light-emitting device with the composite AgNWs/AZO electrode and AgNWs-only electrode. The thickness of AZO was 6 nm. It was observed that the green OLED with the AgNWs/AZO composite electrode produces a higher current density than the AgNWs-only reference electrode when the voltage exceeded 9 V, see Fig. 6a. This illustrates that the AZO layer enhanced hole-injection of the AgNWs layer improved the carrier recombination. Figure 6b shows the luminance–voltage characteristics for the two electrodes; the maximum luminance of composite electrode was up to 10,000 cd/m^2^ at 14 V, which was brighter than for the OLED with AgNWs electrode under same voltage. However, the turn-on-voltage, which is defined as the required voltage to obtain 1 cd/m^2^ of light output, was higher than for the AgNWs electrode see Fig. 6b. The reason of this may be the work function of our composite electrode, which causes a bit slightly higher barrier for hole-junction at low current density.

## Conclusions

We have demonstrated that AZO as a modified layer can improve both connectivity and surface roughness in flexible AgNWs electrodes. The best thickness of the AZO modified layer was 6 nm according to our experiments. The optimized AgNWs/AZO composite electrode shows a high transmittance of 83% and a low sheet resistance of 8.6 Ω/sq, as well as a smooth surface of 0.31 nm root-mean-square roughness. These are comparable to conventional ITO films. The green organic light-emitting device fabricated, using AgNWs/AZO as transparent conducting electrode, has a better I–V–L characteristic than the AgNWs-only device. These results suggest that the smooth AgNWs/AZO composite electrode can be used for flexible organic light-emitting devices and other flexible devices without loss of optical and electrical performance.

## Abbreviations

AgNWs: Silver nanowires
DEZ: [Zn(C2H5)2]: Diethylzinc
ITO: Indium tin oxide
TMA: Trimethylaluminium
